# Supplementary material for: Circulating miR-122-5p and miR-375 as Potential Biomarkers for Bone Mass Recovery after Parathyroidectomy in Patients with Primary Hyperparathyroidism: A Proof-of-Concept Study
Source: Diagnostics (Basel). 2021 Sep 17;11(9):1704. doi: 10.3390/diagnostics11091704 (PMC8472510; doi:10.3390/diagnostics11091704)
Supplement: Supplementary file 1 [file diagnostics-11-01704-s001.zip › diagnostics-1374614-supplementary.pdf]

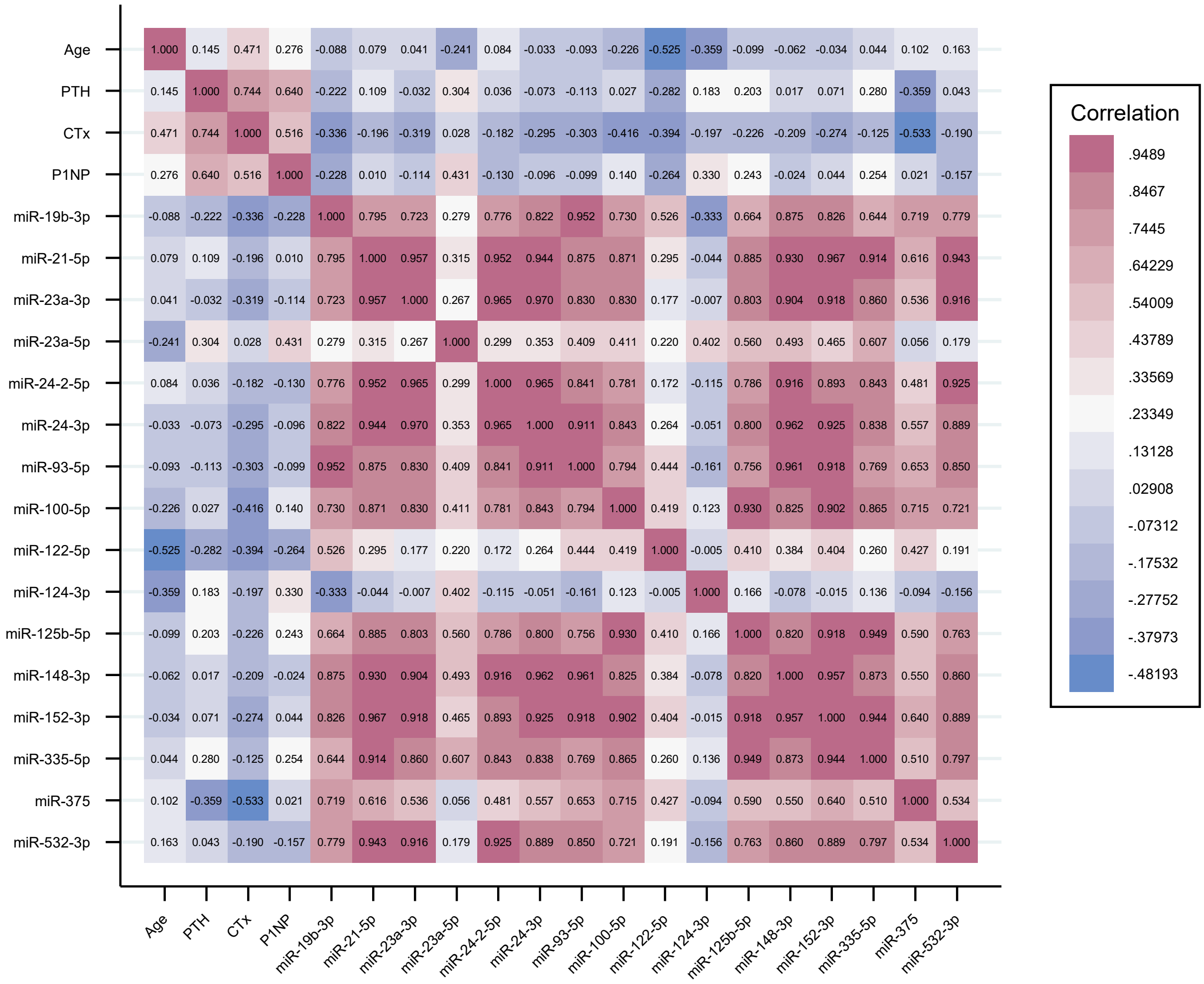

Figure S1. Pearson correlation heatmap of selected osteoporotic fracture-related miRNAs and study covariates. Abbreviations: PTH, parathyroid hormone; CTx, beta-C-terminal telopeptide; P1NP, procollagen type 1 N-terminal propeptide.

Table S1. The pre-specified panel of selected miRNAs and target genes related to osteoporosis and primary hyperparathyroidism.

MiScript Primer Assay (Catalog #Qiagen)	Description	Gene Symbol	Predicted Target Genes	miRNA Sequence
MIMAT0000074	mature miRNA	hsa-miR-19b-3p	AR, IGF1, MAPK1, THBS1	5′ UGUGCAAAUCCAUGCAAAACUGA
MIMAT0000076	mature miRNA	hsa-miR-21-5p	ABCB1, IL1B, LRP6, MAPK1	5′ UAGCUUAUCAGACUGAUGUUGA
MIMAT0000078	mature miRNA	hsa-miR-23a-3p	BMP2K, CALCR, CNR1, ITGA1, LRP5, PTK2B, RUNX2, STAT1, VDR	5′ AUCACAUUGCCAGGGAUUUCC
MIMAT0004496	mature miRNA	hsa-miR-23a-5p	ALPL, PLOD1	5′ GGGGUUCCUGGGGAUGGGAUUU
MIMAT0004497	mature miRNA	hsa-miR-24-2-5p	-	5′ UGCCUACUGAGCUGAAACACAG
MIMAT0000080	mature miRNA	hsa-miR-24-3p	CALCR, IGF1, IL1A, IL1B, MTHFR, PTHLH, SOST, TNF, *ADM*, *CDC73*, *MEN1*, *NOS3*, *SDHAF2*	5′ UGGCUCAGUUCAGCAGGAACAG
MIMAT0000093	mature miRNA	hsa-miR-93-5p	ANKH, BMP2, BMP2K, CD47, CTSK, ESR1, ESR2, ICAM1, PTHLH, TGFB1, *PTH*	5′ CAAAGUGCUGUUCGUGCAGGUAG
MIMAT0000098	mature miRNA	hsa-miR-100-5p	ESR1, IGF1R	5′ AACCCGUAGAUCCGAACUUGUG
MIMAT0000421	mature miRNA	hsa-miR-122-5p	CA10, IGF1R, MAPK1, RUNX2, TGFB1, VDR	5′ UGGAGUGUGACAAUGGUGUUUG
MIMAT0000422	mature miRNA	hsa-miR-124-3p	AR, BMP2K, **COL1A1**,	5′ UAAGGCACGCGGUGAAUGCC

			FGFBP1, IL6, LRP6, MAPK1, PLOD1, RUNX2, TGFB1, VDR, VKORC1	
MIMAT0000423	mature miRNA	hsa-miR-125b-5p	ANKH, CD44, IGF1R, VDR, *SLC26A6*	5′ UCCCUGAGACCCUAACUUGUGA
MIMAT0000243	mature miRNA	hsa-miR-148-3p	CNR1, ESR1, IGF1, KIT, RUNX2	5′ UCAGUGCACUACAGAACUUUGU
MIMAT0000438	mature miRNA	hsa-miR-152-3p	CNR1, DKK1, ESR1, IGF1R, KIT, RUNX2	5′ UCAGUGCAUGACAGAACUUGG
MIMAT0000765	mature miRNA	hsa-miR-335-5p	PLOD1	5′ UCAAGAGCAAUAACGAAAAAUGU
MIMAT0000728	mature miRNA	hsa-miR-375	KIT, RUNX2	5′ UUUGUUCGUUCGGCUCGCGUGA
MIMAT0004780	mature miRNA	hsa-miR-532-3p	CALCR, **COL1A1**, SPARC, STAT1, THBS1, VDR	5′ CCUCCCACACCCAAGGCUUGCA

Predicted target genes related to osteoporosis are shown in plain text and predicted target genes related to primary hyperparathyroidism are shown in *italics*. Predicted target genes related to both osteoporosis and primary hyperparathyroidism are shown in **bold**.

Table S2. Univariable regression (β coefficient) for the association of log-transformed preoperative miRNAs (−△Ct) and lumbar spine (LS) and femoral neck (FN) bone mineral density (BMD) percent change from baseline (%), 1 year after parathyroidectomy, in PHPT patients.

BMD Percent Change from Baseline (%)	Lumbar Spine		Femur Neck	
	β Coefficient	*p*-Value	β Coefficient	*p*-Value
hsa-miR-19b-3p	0.07	0.929	−0.43	0.786
hsa-miR-21-5p	0.82	0.392	1.16	0.565
hsa-miR-23a-3p	0.55	0.509	0.47	0.788
hsa-miR-23a-5p	2.09	0.147	2.86	0.356
hsa-miR-24-2-5p	0.47	0.495	0.25	0.865
hsa-miR-24-3p	0.43	0.555	0.08	0.961
hsa-miR-93-5p	0.39	0.639	0.5	0.771
hsa-miR-100-5p	0.85	0.326	0.07	0.968
hsa-miR-122-5p	−0.83	0.289	−1.82	0.262
hsa-miR-124-3p	3.22	0.057	0.87	0.821
hsa-miR-125b-5p	1.29	0.289	1.73	0.503
hsa-miR-148-3p	0.58	0.52	0.73	0.701
hsa-miR-152-3p	0.84	0.436	1.52	0.497
hsa-miR-335-5p	1.35	0.192	2.36	0.28
hsa-miR-375	0.24	0.764	−0.66	0.685
hsa-miR-532-3p	0.59	0.545	1.59	0.429
